# Draft Genome Sequences of the Multiresistant *Escherichia coli* C20 Strain, Isolated from Domestic Chicken Gut Microbiota

**DOI:** 10.1128/genomeA.00751-17

**Published:** 2017-08-03

**Authors:** Rong-Chuan Tian, Wei Huang

**Affiliations:** a The Affiliated High School of Fujian Normal University, Fuzhou, Fujian, China; b Institute of Quality Standards and Testing Technology for Agro-Products, Fujian Academy of Agricultural Sciences, Fuzhou, Fujian, China

## Abstract

*Escherichia coli* C20, isolated from domestic chicken gut microbiota, demonstrated multidrug resistance to the tested antibiotics. Here, we present the draft genomic sequences of *E. coli* C20, along with that of its plasmid. The final assembly yielded a chromosome of 4,640,940 bp and plasmid of 277,380 bp, with average coverages of 146.95-fold and 35.63-fold, respectively.

## GENOME ANNOUNCEMENT

*Escherichia coli* is frequently used as a representative commensal or pathogenic bacterium, being widely distributed among different ecological niches (1). When studying antimicrobial resistance in farm animals, *E. coli* isolates are of special concern because of their putative role as a source of antimicrobial resistance determinants that could spread on land and in water, thereby reaching humans indirectly (2–5).

*Escherichia coli* strain C20, isolated from domestic chicken gut microbiota, demonstrated resistance to 7 of 10 tested antibiotics (6). The nucleotide sequence of the *E. coli* C20 genome was sequenced from a paired-end library, with an average insert size of 500 bp using the Illumina HiSeq 2000. The trimmed reads were *de novo* assembled using CLC Genomic Workbench 9.5.2, with default parameters, yielding 386 and 27 contigs with average coverages of 146.95-fold and 35.63-fold, respectively. The contigs were then divided into chromosome and plasmid parts, according to coverage. The chromosome and plasmid contigs were separately aligned with the complete genome of *E. coli* K-12 substrain MG1655 (GenBank accession no. CP014225) and *Salmonella enterica* plasmid pHK0653 (GenBank accession no. KT334335) as references for manual scaffolding in Mauve (7). The gaps within these scaffolds were then filled using GapFiller (8). Functional annotation of final genomic sequences was carried out using RAST (9), and phylogenomic analyses were carried out using the core genomic data from 39 *E. coli-*related genomes produced in MicroScope, according to the methods described by Ma et al. (10). The resistance genes were annotated in the Comprehensive Antibiotic Resistance Database (CARD) online server to predict the potential antibiotic resistance determinants in *E. coli* C20 (11).

The final assembly of C20 genomic DNA sequences yielded a chromosome of 4,640,940 bp and plasmid of 277,380 bp, with 45 and 4 superscaffolds and average GC contents of 50.8% and 46.4%, respectively. The C20 chromosome contained 4,389 protein-coding sequences, 87 tRNA genes, and 7 complete rRNA loci. The C20 plasmid harbored 350 coding sequences. Altogether, 154 antibiotic resistance determinants, belonging to 9 different resistance gene families, were annotated for the *E. coli* C20 genome in the CARD website, including 7 resistance genes in the chromosome and 147 in C20 plasmid. Among the previously identified 7 antibiotic resistance genes in the multidrug-resistant strain *E. coli* C20, only the genes conferring resistance to fluoroquinolone antibiotics, including nalidixic acid and ciprofloxacin, were identified in the chromosome; the other 5 resistance genes were found to be expressed in the plasmid (6, 12). Phylogenomic analysis of *E. coli* C20 and 39 other *E. coli*-related genomes based on 2,055 core genes clustered multidrug-resistant strain *E. coli* C20 into the *E. coli* K-12 group, which also includes substrains DH1 (ME8569), BW2952 (MC4100), and MG1655. The nucleotide sequences of the four *E. coli* K-12 genomes shared up to 99 to 100% identity. However, no plasmid sequence was reported and published for K-12 substrains DH1, BW2952, and MG1655. The *E. coli* C20 plasmid showed 97% sequence identity with the plasmid pHK0653 from *S. enterica* strain ST06-53. The results demonstrate that the chicken gut bacterial strain *E. coli* C20 mainly acquired resistance by transfer of the plasmid from the environmental microbiome.

### Accession number(s).

This *E. coli* C20 whole-genome shotgun project has been deposited at GenBank under the accession number NGBR00000000 and consists of sequences NGBR01000001 to NGBR01000048.

## References

[1] Marchant M, Moreno MA 2013 Dynamics and diversity of *Escherichia coli* in animals and system management of the manure on a commercial farrow-to-finish pig farm. Appl Environ Microbiol 79:853–859. doi:10.1128/AEM.02866-12. 23160136 PMC3568553

[2] Bailey JK, Pinyon JL, Anantham S, Hall RM 2010 Commensal *Escherichia coli* of healthy humans: a reservoir for antibiotic-resistance determinants. J Med Microbiol 59:1331–1339. doi:10.1099/jmm.0.022475-0. 20671087

[3] Andremont A 2003 Commensal flora may play key role in spreading antibiotic resistance. ASM News 69:601–607.

[4] Marshall BM, Ochieng DJ, Levy SB 2009 Commensals: underappreciated reservoir of antibiotic resistance. Microbe 4:231–238. doi:10.1128/microbe.4.231.1.

[5] Aarestrup FM 2005 Veterinary drug usage and antimicrobial resistance in bacteria of animal origin. Basic Clin Pharmacol Toxicol 96:271–281. 15755309 10.1111/j.1742-7843.2005.pto960401.x

[6] Zhou J, Ma R, Li D, Tian R, Li M, Luo Y, Liu P, Tian B 2016 Diversity and distribution of antibiotic resistance for culturable bacteria from domestic poultry. J Fujian Agric Forest Univ (Nat Sci Edit) 45:56–64.

[7] Darling AE, Mau B, Perna NT 2010 ProgressiveMauve: multiple genome alignment with gene gain, loss and rearrangement. PLoS One 5:e11147. doi:10.1371/journal.pone.0011147. 20593022 PMC2892488

[8] Nadalin F, Vezzi F, Policriti A 2012 GapFiller: a *de novo* assembly approach to fill the gap within paired reads. BMC Bioinformatics 13:8. doi:10.1186/1471-2105-13-S14-S8. 23095524 PMC3439727

[9] Aziz RK, Bartels D, Best AA, DeJongh M, Disz T, Edwards RA, Formsma K, Gerdes S, Glass EM, Kubal M, Meyer F, Olsen GJ, Olson R, Osterman AL, Overbeek RA, McNeil LK, Paarmann D, Paczian T, Parrello B, Pusch GD, Reich C, Stevens R, Vassieva O, Vonstein V, Wilke A, Zagnitko O 2008 The RAST server: rapid annotations using subsystems technology. BMC Genomics 9:75. doi:10.1186/1471-2164-9-75. 18261238 PMC2265698

[10] Ma RQ, Cao Y, Cheng ZQ, Lei SN, Huang W, Li X, Song YK, Tian BY 2017 Identification and genomic analysis of antifungal property of a tomato root endophyte *Pseudomonas* sp. p21. Antonie Van Leeuwenhoek 110:387–397. doi:10.1007/s10482-016-0811-5. 28000056

[11] McArthur AG, Waglechner N, Nizam F, Yan A, Azad MA, Baylay AJ, Bhullar K, Canova MJ, De Pascale G, Ejim L, Kalan L, King AM, Koteva K, Morar M, Mulvey MR, O’Brien JS, Pawlowski AC, Piddock LJ, Spanogiannopoulos P, Sutherland AD, Tang I, Taylor PL, Thaker M, Wang W, Yan M, Yu T, Wright GD 2013 The Comprehensive Antibiotic Resistance Database. Antimicrob Agents Chemother 57:3348–3357. doi:10.1128/AAC.00419-13. 23650175 PMC3697360

[12] Huang K, Xu CW, Zeng B, Xia QQ, Zhang AY, Lei CW, Guan ZB, Cheng H, Wang HN 2014 Dynamics of quinolone resistance in fecal *Escherichia coli* of finishing pigs after ciprofloxacin administration. J Vet Med Sci 76:1213–1218. doi:10.1292/jvms.14-0025. 24919413 PMC4197147

